# O06 Development of AWaRe antibiotic quality indicators for optimal use

**DOI:** 10.1093/jacamr/dlaf230.006

**Published:** 2025-12-04

**Authors:** Annie Heath, Jan Goelen, Pem Chuki, Aislinn Cook, Filip Djukic, Nga Thi Thuy Do, Elisa Funiciello, Sumanth Gaundra, Brian Godman, Yara Mohsen Khalaf, Guilia Lorenzetti, Marc Mendelson, Catrin E Moore, Claudia G S Osorio-de-Castro, Zikria Saleem, Jeroen Schouten, Elizabeth Tayler, Evelyn Wesangula, A W aR e-B ased Q I s Delphi Panel, Stephen M Campbell, Michael Sharland

**Affiliations:** Centre for Neonatal and Paediatric Infection, Institute for Infection and Immunity, City St. George’s University of London, London, UK; Centre for Neonatal and Paediatric Infection, Institute for Infection and Immunity, City St. George’s University of London, London, UK; Antimicrobial Stewardship Unit, Jigme Dorji Wangchuck National Referral Hospital, Thimphu, Bhutan; Centre for Neonatal and Paediatric Infection, Institute for Infection and Immunity, City St. George’s University of London, London, UK; Centre for Neonatal and Paediatric Infection, Institute for Infection and Immunity, City St. George’s University of London, London, UK; Oxford University Clinical Research Unit, Hanoi, Vietnam; Centre for Neonatal and Paediatric Infection, Institute for Infection and Immunity, City St. George’s University of London, London, UK; Washington University School of Medicine, Saint Louis, MO, USA; Strathclyde Institute of Pharmacy and Biomedical Sciences, Strathclyde University, Glasgow, UK; Department of Epidemiology, High Institute of Public Health, Alexandria, Egypt; Centre for Neonatal and Paediatric Infection, Institute for Infection and Immunity, City St. George’s University of London, London, UK; Division of Infectious Diseases and HIV Medicine, Department of Medicine, Groote Schuur Hospital, University of Cape Town, Cape Town, South Africa; Centre for Neonatal and Paediatric Infection, Institute for Infection and Immunity, City St. George’s University of London, London, UK; Sergio Arouca National School of Public Health, Rio De Janeiro, Brazil; Department of Pharmacy Practice, College of Pharmacy, Qassim University, Buraydah, Qassim, Saudi Arabia; Radboudumc Community of Infectious Diseases, Radboudumc, Nijmegen, The Netherlands; Centre for Neonatal and Paediatric Infection, Institute for Infection and Immunity, City St. George’s University of London, London, UK; East Central and Southern Africa Health Community, Arusha, United Republic of Tanzania; School of Health Sciences, University of Manchester, Manchester, UK; Centre for Neonatal and Paediatric Infection, Institute for Infection and Immunity, City St. George’s University of London, London, UK

## Abstract

**Background:**

The use of antibiotics varies by setting—hospital, outpatient/primary care—with a disproportionate impact of antimicrobial resistance in low-and middle-income countries. The WHO AWaRe (Access/Watch/Reserve) book gives detailed guidance on the optimal use of antibiotics across primary care and hospitals for adults and children with the aim of improving the quality of use of essential antibiotics.

**Objectives:**

To develop universally applicable, model sets of appropriate and feasible quality indicators based on the WHO AWaRe system for primary care, hospital and general indicators for optimal antibiotic use.

**Methods:**

Indicators identified in a scoping review were revised to focus on primary care and hospital facility infections in the AWaRe book. Condition-specific indicators captured measures such as appropriate antibiotic use, total daily dose and proportion of Access or Watch antibiotics. General indicators were developed from prescribing and dispensing guidelines in the AWaRe book and covered themes from proper documentation in medical records to population-level antibiotic use. The indicators were evaluated through a two-round Global Delphi Technique of 104 and 107 panellists, respectively, to determine appropriateness and feasibility in national and local settings, followed by a two-round RAND/UCLA Appropriateness Method (RAND/UCLA) with 12 panellists rating indicators on a global scale. Panels comprised experts from every WHO region. In Round 1 of each method, panellists also rated clarity and could suggest rewording or new indicators. Findings from Round 2 are reported. The median rating was calculated for each indicator to determine agreement. A median of 7–9 was considered appropriate or feasible. If ≥80% of panellists rated within ±1 of the 7–9 median, the indicator was considered appropriate with agreement.

**Results:**

The indicators covered nine clinical conditions in primary care and nine in hospital settings, including respiratory tract infections, diarrhoea, urinary tract infections (UTIs), sepsis and others. There were 102 indicators (Primary Care: 46; Hospital: 39; General: 17) in Round 2 of the Delphi Technique. Of these, 100% were rated appropriate and 99 (97.1%) feasible in a local context. During the RAND/UCLA method, 136 indicators (Primary Care: 56; Hospital: 60; General: 20) were rated in Round 2, with 131 (96.3%) appropriate and 72 (52.9%) feasible in a global context. From these broad sets, 12 indicators from the Delphi Technique and 31 indicators from the RAND/UCLA method were rated both appropriate and feasible with agreement respectively. Most indicators rated appropriate and feasible with agreement by the Delphi panel measured the proportion of patients receiving Access or Watch antibiotics across clinical conditions. Those rated appropriate and feasible with agreement by the RAND/UCLA panel measured appropriate antibiotic choice, dose, duration and the proportion of Access and Watch antibiotics

**Conclusions:**

These model AWaRe-based, universally applicable quality indicators can be locally adapted and measured with different tools to improve the optimal use of antibiotics and inform global and country specific antimicrobial stewardship programmes (AMS).

